# How prepared are young, rural women in India to address their sexual and reproductive health needs? a cross-sectional assessment of youth in Jharkhand

**DOI:** 10.1186/s12978-015-0086-8

**Published:** 2015-10-17

**Authors:** Sushanta K. Banerjee, Kathryn L. Andersen, Janardan Warvadekar, Paramita Aich, Amit Rawat, Bimla Upadhyay

**Affiliations:** Ipas Development Foundation, E-63 Vasant Marg, Vasant Vihar, New Delhi 110 057 India; Ipas, 300 Market St., Suite 200, Chapel Hill, NC 27516 USA; Ipas Development Foundation, C-218 Ashok Path Road #2, Ashok Nagar, Ranchi, 834 002 Jharkhand India

**Keywords:** Young women, Sexual health, Reproductive health, Abortion, India, Health services, Agency

## Abstract

**Background:**

Young, rural Indian women lack sexual and reproductive health (SRH) information and agency and are at risk of negative sexual and reproductive health outcomes. Youth-focused interventions have been shown to improve agency and self-efficacy of young women to make decisions regarding their sexual and reproductive health. The objectives of this study were to assess young women’s sexual and reproductive health knowledge; describe their health-seeking behaviors; describe young women’s experiences with sexual and reproductive health issues, including unwanted pregnancy and abortion; and identify sources of information, including media sources.

**Method:**

A cross-sectional survey with a representative sample of 1381 married and unmarried women young women (15–24 years) from three rural community development blocks in Jharkhand, India was conducted in 2012. Participants were asked a series of questions related to their SRH knowledge and behavior, as well as questions related to their agency in several domains related to self-efficacy and decision-making. Linear regression was used to assess factors associated with greater or less individual agency and to determine differences in SRH knowledge and behavior between married and unmarried women.

**Results:**

Despite national policies, participants married young (mean 15.7 years) and bore children early (53 % with first birth by 17 years). Women achieved low composite scores on knowledge around sex and pregnancy, contraception, and abortion knowledge. Around 3 % of married young women reported experiencing induced abortion; 92 % of these women used private or illegal providers. Married and unmarried women also had limited agency in decision-making, freedom of mobility, self-efficacy, and financial resources. Most of the women in the sample received SRH information by word of mouth.

**Conclusions:**

Lack of knowledge about sexual and reproductive health in this context indicates that young rural Indian women would benefit from a youth-friendly SRH intervention to improve the women’s self-efficacy and decision-making capacity regarding their own health. A communication intervention using outreach workers may be a successful method for delivering this intervention.

## Background

In India, young women, particularly those living in rural areas, are at high risk for negative sexual and reproductive health (SRH) outcomes, with those ages 15–24 years accounting for 41 % of total maternal deaths in India [1]. Early marriage, combined with lack of SRH knowledge and information, and limited agency to negotiate sexual encounters contribute to early and unprotected sex for youth [2, 3]. Despite multiple Indian policies aimed at delaying marriage [4–7], nearly half of women 20–24 years (47 %) report marrying before the legal age of 18 [8]. Given the additional social pressure of proving fertility, it is not surprising that 30 % of women in India give birth before age 18, and 53 % do so by age 20 [8].

Although evidence regarding unintended pregnancies and abortion among youth is limited in India, one study suggests that as much as 41 % of all abortions are among young women [8, 9]. Unsafe abortion accounts for 8-9 % of all maternal deaths in India [1], and given young women’s tendency to approach unskilled and illegal abortion providers, to seek abortion care later in pregnancy, and to delay seeking care for abortion-related complications, the proportion of maternal death due to unsafe abortion is likely higher in young women [10–13].

Young women continue to lack SRH, and particularly abortion, knowledge. Communication campaigns intended to address reproductive health issues often fail to include information about unsafe abortion, or do not reach young women [14]. Additionally, Indian youth may lack sources of SRH information; a recent assessment in Bihar and Jharkhand revealed that youth are apprehensive and unlikely to discuss sensitive SRH issues, including abortion, with older counterparts due to stigma around youth sexuality [15]. In this environment, agency, defined as one’s ability to exercise strategic life choices through personal competence and self-efficacy, can directly influence young people’s sexual and reproductive lives but may be low given the lack of supportive factors [15–17]. Agency enables youth to exercise their preference in the timing of marriage and choice of partner, to make health-related decisions, to access health services and to decide whether and when to engage in sexual relations and contraception [18].

Youth-focused interventions are an important way to address the SRH information and service delivery needs of young women. We conducted a cross-sectional household survey in late 2012 to help inform a youth-focused communication intervention to educate rural young women in India about SRH issues, including safe abortion.

## Methods

Using a cross-sectional survey conducted in August and September 2012, we sought to characterize the sexual and reproductive health knowledge, attitudes and skills of young (age 15–24 years) married and unmarried women in three rural community development blocks (Deoghar, Bagodar and Madhupur) in the Jharkhand State in India. Rural areas in India are grouped into community development blocks that are targeted for development in health, education and communication [19]. Specific objectives of the study were to:

- Describe young women’s experiences with unwanted pregnancy and abortion;
- Identify young women’s sources of SRH information, including media sources;
- Assess young women’s SRH knowledge, including of sex and pregnancy, contraception and legal, safe abortion;
- Describe the health-seeking behaviors of young women, including agency and self-efficacy.

This study underwent ethical review and was approved by the Institutional Review Board of the Centre for Media Studies in New Delhi, India and Allendale Institutional Review Board in the United States.

### Sample

A two-stage sampling strategy was used to select a representative sample of young married and unmarried women from the three blocks. In stage one, 23 villages from each block were selected using probability proportional to size sampling [20]. Households with eligible participants were selected through systematic random sampling from a detailed household listing prepared for each included village. Only one eligible respondent was selected per household; in households with more than one eligible respondent, the Kish table was used to select a study participant [21]. Study participants were recruited and interviewed at their homes by trained study investigators.

### Data collection

All women gave informed consent to participate in the study; for unmarried women aged 15–17 years, parental consent was also obtained. To promote study participant comfort during interviews, young female data collectors were recruited from the study state, and underwent extensive training in study procedures, ethical issues, informed consent, and privacy. Training included classroom sessions, role-play, mock interviews and field practice in similar villages not included in this study sample. Trained data collectors privately administered one of two survey instruments--either for married or unmarried women--to study participants. Instruments were based on previous youth questionnaires [2, 22] and were translated into Hindi and local dialects and field-tested prior to use. Both survey instruments gathered information about respondents’ demographic and socio-economic status, health-seeking behavior and service utilization, knowledge about SRH including comprehensive abortion care (CAC), exposure to information about SRH and CAC including media exposure, and individual agency and self-efficacy. The instrument for married women also included questions related to reproductive history and pregnancy outcome. Married youth reporting an abortion answered an additional module about their abortion experience. This module included questions about the abortion information received from providers or other actors, type of abortion provider and procedure received, and complications.

To ensure privacy and confidentiality, each respondent was asked to choose a private room or other location at or near their household where they would be comfortable talking about sensitive topics. If it was not possible to conduct the interview with sufficient privacy, the data collector scheduled an appointment with the respondent to conduct the interview at a later date. In addition, an anonymous reporting approach was applied to ask particularly sensitive questions, such as those related to pre-marital sexual exposure, by using sealed envelopes. To ensure confidentiality and privacy, a unique identification number was used to link these sealed responses to participants’ questionnaires.

### Data analysis and measures

Descriptive statistics are reported for both categorical (frequency and percentage or median and range) and continuous (mean and standard deviation) variables. Economic status of each participant was generated from a standard of living index based on ownership of durable household goods and assets; higher standard of living indicates greater income and access to modern amenities [23].

To assess young women’s overall knowledge of SRH, participants answered knowledge-based questions in three SRH domains: sex and pregnancy, contraception, and legal aspects of safe abortion. A knowledge score was generated for each domain (0–6 for sex and pregnancy, 0–8 for contraception, and 0–5 for abortion; higher numbers indicate greater number of correct answers). Linear regression was used to determine factors associated with higher knowledge scores.

A composite index measuring agency was generated based on women’s responses to questions in four domains associated with individual agency: decision-making, access to financial resources, freedom of mobility and self-efficacy [24]. We asked participants if they make decisions on their own, jointly with others or if they had no role in decision-making (decision-making), if they have an account or deposit at any bank, post-office or group deposit scheme (access to financial resources), if they are allowed to visit friends, programs, or establishments inside and outside of the village without accompaniment (freedom of mobility) and if they are able to express their opinions, initiate discussion about SRH issues, and refuse sex (self-efficacy), among other questions. Linear regression was used to assess factors associated with greater or less individual agency.

## Results

### Sample demographics

We interviewed 690 married and 691 unmarried women (total *n* = 1381); the overall study response rate was 88 %. Table 1 presents the demographic characteristics of the study participants. Only 12 % of unmarried women included in the study were over the age of 18 and average age at marriage reported in the married women group was 15.7 years (SD 1.7). Despite their overall younger age, women in the unmarried group reported greater educational attainment than those in the married group: 83 % of unmarried reported having an education at middle or higher level, compared to only 38 % of married women. Over 85 % of the young women fell into the low and moderate standard of living category and belonged to castes traditionally associated with low socioeconomic status (Scheduled Caste, Scheduled Tribe and Other Backward Class).

**Table 1:** Socio economic profile of married and unmarried young women in Jharkhand, 2012

	Married		Unmarried		Total	
	(*n* = 690)		(*n* = 691)		(*n* = 1381)	
	*n*	%	*n*	%	*n*	%
Current age (in years)						
15–17	147	(21)	608	(88)	755	(55)
18–20	291	(42)	69	(10)	360	(26)
21–24	252	(37)	14	(2)	266	(19)
Age, in years						
Mean	19.5		15.9		17.7	
(sd)	(2.26)		(1.43)		(2.60)	
Age at marriage, in years						
Mean	15.7					
(sd)	(1.69)					
Education						
Never Attended School	231	(34)	63	(9)	294	(21)
Primary	128	(19)	54	(8)	182	(13)
Middle	243	(25)	452	(65)	695	(50)
Secondary & above	88	(13)	122	(18)	210	(15)
Years of schooling^1^						
Mean	6.5		7.8		7.2	
(sd)	(3.10)		(2.37)		(2.76)	
Currently studying						
Yes	35	(5)	463	(67)	498	(36)
No	655	(95)	228	(33)	883	(64)
Religion						
Hindu	501	(73)	471	(68)	972	(70)
Muslim	187	(27)	214	(31)	401	(29)
Other	2	(<1)	6	(1)	8	(1)
Caste						
Scheduled Caste (SC)	98	(14)	86	(12)	184	(13)
Scheduled Tribe (ST)	27	(4)	33	(5)	60	(4)
Other Backward Class (OBC)	476	(69)	463	(67)	939	(68)
General	89	(13)	109	(16)	198	(14)
Type of Family						
Nuclear Family	147	(21)	323	(47)	470	(34)
Joint-extended Family	543	(79)	368	(53)	911	(66)
Types of Occupation						
Farming (Family land)	26	(4)	7	(1)	33	(2)
Agricultural labor	30	(4)	27	(4)	57	(4)
Non-agricultural wage labor	28	(4)	14	(2)	42	(3)
Business & salaried	18	(3)	13	(2)	31	(2)
Not Working	588	(85)	630	(91)	1218	(88)
Standard of Living Index						
Low	321	(47)	307	(44)	628	(46)
Medium	277	(40)	282	(41)	559	(41)
High	92	(13)	102	(15)	194	(14)
Exposed to mass media/ social networks^2^						
TV	412	(60)	487	(70)	899	(65)
Newspaper	145	(21)	370	(54)	515	(37)
Cinema	284	(41)	351	(51)	624	(45)
Radio	72	(11)	127	(18)	199	(14)
Internet	4	(1)	9	(1)	13	(1)
Facebook	3	(<1)	2	(<1)	5	(<1)
Youth Club	2	(<1)	10	(1)	12	(1)
Any Mass Media^3^	445	(65)	551	(80)	996	(72)
Utilization of SRH Services						
Pubic services	147	(21)	75	(10)	222	(16)
Private clinic	436	(63)	283	(41)	719	(52)
Pharmacy/chemist	32	(5)	47	(7)	79	(6)
Traditional healers	226	(33)	292	(42)	518	(38)
Never used any services	38	(6)	108	(16)	146	(11)

^1^Average schooling is for those who attended school

^2^Includes regular and occasional exposure to any medium

^3^Mass Media includes TV, Radio, Newspaper and Movies

A majority of participants (52 %) reported seeking SRH treatment or advice from a private facility or provider. Thirty-eight percent also reported seeking SRH services from a traditional healer or unqualified rural medical practitioner.

### SRH history (among married women)

Reproductive and pregnancy history was gathered only from young married women (Table 2); 82 % had ever been pregnant, and 19 % were pregnant at the time of the study. Early pregnancy was common: 53 % of married women 15–17 had experienced at least one pregnancy, increasing to 83 and 97 % for women 18–20 and 21–24 respectively (data not shown). Approximately 1 in 6 pregnancies were reported by women to be unwanted or mistimed (data not shown); 23 women (3 %) ever had an induced abortion. Contraceptive prevalence was low among young married women not pregnant at the time of the survey (18 %); female sterilization was the most common modern contraceptive used (9 %), followed by condoms (2 %) and pills (2 %). Around four percent of young married women reported using traditional methods.

**Table 2:** Reproductive history among young married women (*n* = 690), Jharkhand 2012

Pregnancy History	Number	Percent	Mean	(sd)
Ever pregnant	563	(82)		
Number of lifetime pregnancies^1^			2.0	(1.15)
Currently pregnant	129	(19)		
Number of live births^1^			1.5	(1.09)
0	210	(30)		
1	238	(35)		
2	159	(23)		
3 or more	83	(12)		
Number of surviving children			1.4	(0.91)
0	216	(31)		
1	255	(37)		
2	160	(23)		
3 or more	59	(9)		
Any pregnancy loss	141	(20)		
Number of pregnancy losses^1^			0.3	(0.60)
Ever had still birth (one or more)	37	(5)		
Number of still births^1^			0.07	(0.29)
Ever had a miscarriage	90	(13)		
Number of miscarriages^1^			0.2	(0.49)
Ever induced abortion	23	(3)		
Number of induced abortions^1^			0.05	(0.26)
Contraception				
Current use^2^	98	(18)		
Method-mix of contraception (among users)				
Female sterilization	52	(9)		
Oral contraceptive pill	10	(2)		
Condom	11	(2)		
IUCD	1	(<1)		
Traditional method (Safe & withdrawal)	24	(4)		

^1^Means and percentages are calculated for young women who had ever been pregnant

^2^Percentage computed among 561 women who are currently not pregnant

Of the 23 married women reporting an induced abortion, only 4 % sought treatment from government facilities (where services are virtually free and provided by trained providers). Instead, most (52 %) went to a private facility, or to a chemist/rural practitioner (43 %); few private facilities are approved to provide abortion by the government, and chemists/rural practitioners are not legally allowed to provide induced abortion in India. Reliance on private sector was also observed for the 90 women who reported a spontaneous abortion (data not shown).

### Sources of SRH information

Women report high exposure to mass media (65 %), with television (65 %), cinema (45 %) and newspaper (37 %) being the most common media formats (Table 1). Very few women in this sample had access to the internet (0.5 % of married and 1.3 % of unmarried women), and even fewer (less than 1 %) women had access to social networking. Youth clubs, common in urban populations, were largely unknown in these rural communities.

Married women were more likely to have received information on SRH, contraception and abortion than unmarried women (Table 3). Among those who did receive any information, both married and unmarried women reported family and friends as the primary source for all three types of information (71 vs 79 %, respectively). Besides family and friends, married women were more likely to have received information from outreach, whereas unmarried more likely to obtain information from mass media. Receiving any abortion information was low for both groups, with unmarried women being significantly less likely to receive any abortion-related information compared to married women (3 vs. 10 %, *p*-val = 0.001). Importantly, a large proportion of women deny having received any information about SRH issues (29 %), contraception (19 %) or abortion (93 %), while 94 % of young women received no information on all three topics (data not shown).

### SRH knowledge

Knowledge about SRH issues was extremely limited in both groups, although married women knew more than unmarried women in all three knowledge categories (Table 4). Of note, 55 % of young married and 71 % of young unmarried women could not correctly respond to any of the five questions about safe abortion. Furthermore, 10 % erroneously believed that abortion is not legal in India (data not shown). Linear regression modeling indicated that, after adjusting for other demographic variables, married women and women with middle or secondary education were more likely to have accurate knowledge in all three knowledge domains (*p* < 0.001), older women (19–24 years) had a better understanding of sex and pregnancy and contraception than younger women (*p* < 0.001), and women with a high standard of living had better knowledge about contraception and abortion (*p* < 0.001). Exposure to any SRH information from any source was found to have strong association with the knowledge level of all three SRH related issues (data not shown).

**Table 3:** Percentage distribution of sources of information on SRH, contraception, and abortion in Jharkhand, 2012

	SRH Information			Contraception Information			Abortion Information		
	Married	Unmarried		Married	Unmarried		Married	Unmarried	
	(*n* = 690)	(*n* = 691)	*p*-value^1^	(*n* = 690)	(*n* = 691)	*p*-value	(*n* = 690)	(*n* = 691)	*p*-value
Received any information	89	69	0.001	89	74	0.001	10	3	0.001
Source of Information^2^									
Friends / neighbors	71	79	0.004	80	82	0.485	73	75	0.809
Family members/ relatives	72	79	0.022	74	72	0.344	42	38	0.697
Husband	34	--		38	--		17	--	
Outreach (AWW/ASHA)	27	15	<0.001	29	14	<0.001	20	17	0.699
Mass media	17	20	0.185	22	37	<0.001	13	42	0.003
Wall Sign	2	10	<0.001	9	20	<0.001	1	8	0.100
ANM/ Nurse	5	3	0.069	6	3	0.016	9	4	0.468
Health facility-Public	1	2	0.118	3	1	0.021	3	8	0.258
Health facility-Private	7	2	<0.001	5	1	<0.001	19	0	0.022
Other	1	1	0.963	1	<1	0.156	1	0	0.553

*AWW* Anganwadi worker, *ASHA* Accredited social health activist, *ANM* Auxiliary nurse midwife

^1^P-value associated with Z-test of two 2 sample proportions

^2^Percentage computed among women who reported receiving any information on each topic

**Table 4:** Knowledge of sex/pregnancy, contraception and abortion among married and unmarried young women in Jharkhand, 2012

	Married (*n* = 690)		Unmarried (*n* = 691)		
	*N*	(%)	*n*	(%)	*p*-value^1^
Knowledge of Sex and pregnancy					
No correct response	18	(3)	99	(14)	
1-2 correct responses	244	(35)	449	(65)	
3-4 correct responses	370	(54)	139	(20)	
5 & above	58	(8)	4	(1)	
Composite Score [Range 0–6]					
Mean	2.9		1.8		<0.001
(sd)	(1.24)		(1.07)		
Knowledge of contraception					
No correct response	0	(0)	4	(1)	
1–2 correct responses	261	(38)	563	(82)	
3–4 correct responses	322	(47)	120	(17)	
5 & above	107	(16)	4	(1)	
Composite Score [Range 0–8]					
Mean	3.0		1.7		<0.001
(sd)	(1.45)		(0.89)		
Knowledge of legal aspect of safe abortion					
No correct response	382	(55)	488	(71)	
1–2 correct responses	274	(40)	186	(27)	
3–4 correct responses	31	(5)	15	(2)	
5 correct responses	3	(<1)	2	(<1)	
Composite Score [Range 0–5]					
Mean	0.7		0.4		<0.001
(sd)	(0.90)		(0.75)		

^1^P-value associated with Z-test of two sample proportions

### Agency

Irrespective of marital status, young rural women displayed limited power in key domains of agency. Ninety-two percent of married and 99 % of unmarried women had no say in their own health care, while more than 94 % of young women reported no ability to choose any doctor for their own health problem (Table 5). Only 9 % of married and 3 % of unmarried women reported their ability to influence timing of pregnancy or marriage, respectively. Young women did report ability to choose their friends, (married 71 %, unmarried 82 %, but few were involved in making decisions about spending money (married 20 %, unmarried 26 %) and buying their own clothes (married 13 %, unmarried 17 %).

**Table 5:** Young women’s measures of agency: Participation in decision-making, ability to visit alone and self-efficacy and composite agency scores by marital status, Jharkhand 2012

	Married		Unmarried		
	(*n* = 690)		(*n* = 691)		
	*n*	%	*n*	%	*p*-value^1^
Participation in decision-making					
Choosing a friend		(71)		(82)	<0.001
Spending money		(22)		(26)	0.061
Buying cloths for own		(17)		(18)	0.530
Own health care		(8)		(1)	<0.001
Choosing any doctor		(6)		(1)	<0.001
When to get pregnant		(9)		n/a	--
When to get married		n/a		(3)	--
Mobility					
Able to visit program inside village alone	96	(14)	106	(15)	0.453
Able to visit shop inside village alone	174	(25)	359	(52)	<0.001
Able to visit friend inside village alone	172	(25)	318	(46)	<0.001
Able to visit program outside village alone	20	(3)	23	(3)	0.646
Able to visit shop outside village alone	26	(4)	23	(3)	0.658
Able to visit friend outside village alone	14	(2)	14	(2)	0.997
Able to visit doctor alone	12	(2)	4	(1)	0.044
Self-efficacy					
No difficulty expressing opinion to elders	163	(24)	159	(23)	0.787
Can talk confidently to a provider on SRH issues including abortion	153	(22)	103	(15)	0.001
Can initiate discussing issues related to SRH with my friends	217	(31)	141	(20)	<0.001
Can help my friends to choose a trained doctor who provides abortion	146	(21)	47	(7)	<0.001
Able to say “no” to sex if I don’t feel like having sex	238	(35)	--	--	--
Composite agency scores					
	Mean	(sd)	Mean	(sd)	
Decision making [Range: 0–12]	2.5	(2.25)	2.6	(1.79)	0.372
Choice of mobility [Range: 0–12]	6.6	(1.93)	7.5	(1.67)	<0.001
Access to money [Range: 0–3]	0.2	(0.63)	0.1	(0.56)	0.005
Sense of self-worth [Range: 0–4]	0.9	(1.15)	0.6	(0.89)	<0.001
Overall Agency [Range: 0–33]	10.2	(3.99)	10.8	(3.06)	0.005

^1^P-value associated with Z-test of two sample proportions

Autonomy to save money was uniformly limited, only 6 % young women reported having a bank account (5 % independently, without a spouse or parent or other relatives; data not shown). Both married and unmarried women were highly restricted in their mobility outside the village and ability to visit doctors alone (Table 5); married participants were also significantly more restricted in their mobility within the village, when compared with unmarried participants (visiting a friend inside village: 25 vs 46 %, *p* = 0.001; visiting a shop inside village: 25 vs 52 %, *p* = 0.001). Both married and unmarried young women reported limited self-efficacy in expressing their own opinions, discussing SRH issues and helping a friend to choose a trained abortion provider. Of note, 65 % of young married women reported that they are unable to refuse sex with their spouse.

Calculated from these domains, the mean composite score of agency was less than 11 out of a possible 33, with no difference seen between married and unmarried young women. Scores were low in all four domains. However, married women scored higher on sense of self-worth (*P* < 0.001) and access to money (*p* < 0.01) and lower on choice of mobility (*p* < 0.001) as compared to unmarried women (Table 5). Scores were low in all four domains. The results of the multivariate analyses reveal age (19–24 years), education (secondary and higher), high standard of living and exposure to mass media are associated with a higher sense of agency (*p* < 0.001), while young women from joint/extended families and non-Hindu religion (*p* < 0.000) were more likely to express limited agency (data not shown).

## Discussion

Many of our findings underscore the limitations of young women’s knowledge and agency around sexual and reproductive health in rural India. Despite multiple national policies against early marriage and promoting universal education, many women in our sample were married before age 18 and many young women had limited education, particularly those who were married. Reproductive histories of young women clearly reflect the continuing trends of early pregnancy and high fertility. More than one-third of young married women reported two and more surviving children; one-fifth of young married women had experienced at least one pregnancy loss. In contrast to urban youth, almost one-tenth of rural young women reported completing desired family size and had accepted female sterilization as contraception. Even after six decades of official family planning in India, young women rarely accept modern methods, including condom, oral contraceptive pills or intrauterine contraceptive devices, as shown in our study and others [2]. Moreover, after four decades of legal abortion in India (MTP Act), almost all young women were unaware that abortion is legal.

The participants demonstrated very limited agency to make decisions around strategically important issues such as age of marriage or when to have children, freedom of movement, access to financial resources and self-efficacy. However, spousal control over their wife’s mobility inside the village and decision-making appears to be stricter than parental control over their unmarried daughters’ mobility and decision-making. The married women in our study also had little control over when to have sex. Our findings on agency are consistent with similar work in India, an indication of the enduring gender imbalance that prevents young women from accessing necessary SRH information and services [2, 17].

These data provide a foundation for the development of an SRH intervention tailored to young women in rural India. Effective SRH programs for young women should include strategies that build agency and life skills in young women, improve male involvement through targeted male education and gender-sensitization, improve support from stakeholder adults in the community through education of parents and guardians, and facilitate an overall improvement in communication around and access to SRH knowledge and services [25]. In particular, communication campaigns must clearly relay the legally supported reproductive rights of young women, as represented through acts against early marriage and legalization of abortion. Previous interventions have attempted to improve SRH knowledge among young women through school-based campaigns. However, in the Indian context, teacher-led SRH education are heavily debated, as these programs can be challenging to implement due to teachers’ discomfort with the topic lack of skills to deal with the social and psychological implications of engaging students on sensitive topics around sexuality [26–28]. Furthermore, school-based programs are not able to address other key barriers that young women face, including lack of support from spouse or partners, parents and the community at large. Specifically in rural India, studies have shown that community-based programs such as communication campaigns and interventions that deliver health education directly to youth through peer or outreach providers can be effective in improving knowledge and uptake of SRH [29–31]. These interventions not only engage youth directly, but also aim to improve overall community attitudes towards young women’s access to sexual health care. The above evidence, combined with the findings of this study – that show young women receive SRH knowledge through friends and family and also have access to media channels such as television and radio – indicate the potential of reaching youth through a community-based campaign that uses media, outreach workers and peer education to improve young women’s knowledge and agency around SRH, and to create a more supportive community environment for all young women to exercise their reproductive health desires. Special attention should be paid to the unique needs of married versus unmarried women.

Our findings should be viewed within the context of the study’s limitations. Household surveys rely on self-report by the respondents and reporting and recall bias are possible. Like other demographic and social surveys, the incidence of abortion and knowledge of abortion-related information may be under-reported. The findings of this study are based on three selected blocks and cannot be generalized to the entire youth population of Jharkhand; however, most of the findings on young women’s knowledge, attitudes, behavior, and practice are in line with other published research in India [2, 17].

## Conclusion

Irrespective of marital status, rural young women are ill-equipped to deal with their sexual and reproductive health. This assessment of young women’s awareness of SRH matters and current practices of utilizing health-care services for reproductive health issues including abortion and post-abortion complications suggests a need for a comprehensive, youth-focused behavior change communication intervention.

## Abbreviations

CAC: Comprehensive abortion care
SRH: Sexual and reproductive health
